# Medical Titles

**Published:** 1907-06-01

**Authors:** J. Grimshaw

**Affiliations:** Holly Bank Road, Clifton Park, Birkenhead


### Editor's Letter-Box.

[Our Correspondents are reminded that prolixity is a great bar to publication, and that brevity of style and conciseness of statement greatly facilitate early insertion.]

### "MEDICAL TITLES."

Sir,—The annotation on "Medical Titles" is full of spite and altogether objectionable. I happen to be an M.D.(Lond.), and, notwithstanding the disparaging remarks of your contributor, I am rather—I almost wrote, naturally—proud of my possession. I do not deceive myself by thinking that the degree confers upon me ability. I do not even believe that it adds an atom to my professional status among the laity. But I certainly think I should be credited with having passed a certain professional test which my brother practitioners here have not.

While I do not consider myself superior to my medical brethren, *ipso facto,* for being a real, live "doctor," yet I contend that it is wrong for them to pose as such when they are not, and to emulate my position by falsely styling themselves "doctor" on their door-plate or bill-heads. There is no necessity for them to do this, and by doing it they undeniably demonstrate their belief in the advertising value of the title to which they have no academic claim.

My mind may be of a "juvenile order," but for the life of me I cannot see any disgrace in passing "a moderately difficult examination," or any credit due to a licentiate or member for failing or shirking it. On the other hand, my experience teaches me that a very large majority of the latter have pigeon-holed for ready use voluble excuses for not being M.D.s or F.R.C.S.s!

You ask for suggestions. Allow me, Sir, to offer one: award honour where it is due, and do not discount industry and give offence to your readers by disparaging the medical degrees of London University, but rather condemn the advertising methods of those medical men who seek lucrative practices by "plating" claims which are calculated to attract the public, and who prefer to tout for appointments than qualify for them by exams.

Yours, etc., J. Grimshaw.

Holly Bank Road, Clifton Park, Birkenhead.
May 25, 1907.

